# Narrating Loneliness: Isolation, Disaffection, and the Contemporary Novel

**DOI:** 10.1007/s10912-024-09855-z

**Published:** 2024-06-22

**Authors:** Neus Rotger

**Affiliations:** Universitat Oberta de Catalunya (UOC), Barcelona, Spain

**Keywords:** Loneliness, Isolation, Affect, Health narrative, Pandemic, Contemporary novel

## Abstract

This article focuses on the ways in which narrative accounts of loneliness in literature problematize current definitions of this important and yet underexplored determinant of health. I argue that the prevailing conceptualization of loneliness in health research, with a general emphasis on social prescribing, obscures other dimensions of loneliness beyond social connectedness that also need to be accounted for in its definition. Drawing on narrative approaches to health and care and taking as a case study Santiago Lorenzo’s Spanish novel *Los asquerosos* (2018), the article gestures toward a more political—rather than exclusively subjective and relational—reading of loneliness. It shows how the novel’s exploration of loneliness as an ambivalent experience of tranquility and disaffection questions whether there is any direct causation between loneliness and aloneness or social isolation, presenting loneliness not so much as a problem or a social pain in need of curing, but as a symptom of a larger structural crisis. The article also reflects on the ability of literary narratives to illuminate, discuss, and ultimately challenge the underlying dynamics of loneliness, raising questions about how we understand these narratives and the type of agency we attribute to them.

Like past humanitarian emergencies, the COVID-19 crisis has brought to light preexisting shortcomings in public health governance (Horton 2021), making us question how we cope with uncertainty, risk, deprivation, and fear at different scales, and rekindling old debates about freedom and security, individual sovereignty, and public scrutiny. Warnings about the consequences of such health policy failures have focused on the unprecedented measures put in place around the world to prohibit social contact, from strict lockdowns to orders of self-confinement, curfews, quarantines, travel bans, and *cordons sanitaires*. The impact such societal restrictions have had—and continue to have—on pressing social issues related to loneliness has increased awareness about what news headlines have been reporting as “An Epidemic of Loneliness,” “The Loneliness Pandemic,” or loneliness as “the next big global health problem.”^1^ While it is uncertain whether the risk of loneliness has increased due to the pandemic (Ernst et al. 2022), ever since the virus’s outbreak in 2019 more attention has been directed toward loneliness as an important yet overlooked determinant of health.

Recent research shows that loneliness has direct impacts on longevity, mental, and physical health and well-being, especially among vulnerable social groups affected by gender, age-based, ethnic, socioeconomic, and educational inequalities. The adverse effects of loneliness range from depression to increased risks of cardiovascular disease, stroke, diabetes, cognitive decline, and suicide, and its overall influence on early mortality is comparable to other risk factors like smoking, a sedentary lifestyle, and air pollution (Hawkley and Cacioppo 2003; Holt-Lunstad et al. 2015; Leigh-Hunt et al. 2017). Even though the relationship between loneliness and poor health has been well established, researchers, practitioners, and policymakers agree that there is a lack of conceptual clarity around the definition of loneliness, and that more transdisciplinary work is needed to develop a shared language that facilitates the discussion in a global context (Prohaska et al. 2020). This transdisciplinary work requires us to move beyond medical research and explore its connections to other fields in order to address a complex phenomenon that is as much a subjective feeling as it is a collective and therefore political problem.

With the aim of further opening this transdisciplinary discussion, this article focuses on the ways in which narrative accounts of loneliness in literature can help nuance how health scholarship is currently conceptualizing the experience of being and feeling alone. To do so, I draw on recent loneliness research with two main goals in mind: first, to put into dialogue various and sometimes divergent conceptual and methodological approaches to loneliness and related terms such as social isolation, social exclusion, or solitude; and second, to argue that literature and literary studies are uniquely positioned to shed light on this particularly hidden and invisible reality, which might in turn help us develop more effective interventions targeting loneliness and its negative effects. For this, I turn to a reading of Santiago Lorenzo’s *Los asquerosos* (The Loathsome), which became one of the biggest editorial successes in Spain during the first wave of the pandemic, when the lockdown and other social restrictions were particularly severe. Although it was written before the pandemic, Lorenzo’s novel anticipated many of the ills of the current crisis with a story of confinement that compellingly challenges the causation between social isolation and loneliness and invites reflection on how feelings of disaffection can help us imagine new meanings of loneliness, not necessarily negative.

## The politics of loneliness

In a large body of health scholarship, loneliness is defined as a painful subjective feeling that stems from a longing for social connections. Most of these definitions follow the cognitive discrepancy theory put forward in the 1980s by psychologists Daniel Perlman and Laetitia Anne Peplau, who conceptualize loneliness as “a discrepancy between one’s desired and achieved levels of social relations” (1981: 32). This unwelcome feeling of lacking a satisfactory number of meaningful relationships relates to similar—yet not equivalent—experiences of isolation. As much as they are related, there is a distinction between loneliness and social isolation, which refers to the objective state of having a limited social network. However clear the distinction between these terms may be, loneliness and isolation are often enmeshed, which introduces additional confusion into an already elusive phenomenon. Being socially isolated can—but not necessarily does—lead to loneliness; in fact, some studies have found little evidence of the correlation between the two or its impact on health (Jose and Lim 2014; McHugh et al. 2017; Steptoe et al. 2013). This confusion obscures not only that there is a positive and salutary dimension to being alone, as psychiatrist Anthony Storr argued in his classic 1988 study on solitude, but also that there are other reasons beyond social connectedness underlying loneliness that also need to be accounted for in its definition, reasons that point to a more political, rather than exclusively subjective or relational, reading of loneliness.

The focus on social capital, and therefore the stress on helping improve social relations in order to alleviate loneliness, is at the center of a growing line of health interventions that are pushing to eradicate pharmacological treatments to loneliness (Cacioppo et al. 2015; Campbell 2023; Holt-Lunstad 2021; Paquet et al. 2023). These interventions are mostly directed at the elderly, but have also been aimed at young people and other disadvantaged collectives such as persons with disabilities or mental health problems, migrants, asylum seekers, and the LGBTQIA+ community. Based on socialization, participation, community building, and social skills training, these interventions are showing exciting results in clinical trials, although their effectiveness is variable and difficult to determine (Coll-Planas et al. 2017; Gardiner et al. 2018). Despite these varied results, the core idea of the social approach is that loneliness is directly related to the number and quality of connections within a social network, in a manner that appears in clusters and has the potential to spread by contagion, as sociologist Émile Durkheim famously observed about suicide (Cacioppo et al. 2009). This has also been the primary approach taken by governments and public health policies around the world. Ever since the creation of the first Loneliness Ministry in the UK in 2018, governmental strategies have emphasized social prescribing as a means to strengthen the social fabric and thus collectively tackle the problem of loneliness.^2^

In this framing, loneliness is no longer understood from an exclusively biomedical and pharmaceutical perspective. To some extent, though, the idea of loneliness as a disease that needs to be prevented, diagnosed, treated, and ultimately cured persists. One case in point is the abovementioned UK Loneliness Ministry, which spoke about the “risk of loneliness” as “one of the greatest public health challenges of our time,” against which the government’s strategy was positioned as “a vital first step in a national mission to end loneliness” (UK government 2018: 2). The paternalistic undertones of a policy agenda that acknowledges how “government can’t make our friends for us” (3) but invests millions of pounds in helping create “a more connected society” put the focus on a discourse of crisis. This rhetoric of crisis, which is increasingly common in public health literature and government policy (duly amplified in media headlines before and after the pandemic), directs the conversation toward those feeling lonely—the steps *they* need to take to stay connected. As Eleanor Wilkinson has argued, such prevailing narratives function as a way to depoliticize loneliness, because they tend to put the pressure on individuals and communities, thereby concealing a larger structural crisis:

This framing of loneliness becomes a way to circumvent issues of state abandonment: it downplays the ways in which certain bodies are cast out, forced to endure conditions that so often condemn them to isolated lives, that make connections fragile, that grind us down. The language of the “loneliness crisis” masks a series of other crises: rising economic precarity, the dismantling of the welfare state, displacement, systemic racism, the continued dominance of heteropatriarchy. (Wilkinson 2022: 32)

Reflecting on loneliness from the standpoint of feminist and queer studies, Wilkinson makes a compelling argument about how the language of crisis positions loneliness as an individual failure, rather than as a structural condition, and how public health and policy narratives of individualized responsibility often follow a heteronormative logic that positions coupled love, family life, and the community as the best means of alleviating loneliness. Building on the work of Sarah Ahmed (2006, 2010), Lauren Berlant (2011), and others, political readings like Wilkinson’s put the focus on contemporary conditions of loneliness and emphasize how loneliness as a social and cultural construct is in part shaped by normative discourses around aspiration and the pursuit of happiness. Perlman and Peplau’s aforementioned discrepancy theory, which most definitions of loneliness rely on, is still valid under these readings, except the discrepancy stems from a disconnect between neoliberal fantasies of the good or better life and the affects that they produce in us.

It might be time, then, to understand loneliness not so much as a problem or a disease in need of curing but as a symptom of a larger crisis. This change in focus is important because it destabilizes the foundations of an ideology that naturalizes the structural conditions underlying loneliness and that precludes our ability to imagine the possibility of change. Reading loneliness as a symptom and therefore uncovering its political potentialities, as Wilkinson does, means examining how “feelings of disaffection and alienation can help us imagine other worlds” (2022: 35). That is, it means proposing a scenario in which shared loneliness can serve to point the conversation away from individual responsibility to highlight instead the structural violences and inequalities underlying loneliness, thus challenging extended narratives about the inevitability of these—an idea I will turn to later in my reading of Santiago Lorenzo’s novel. The question about loneliness, however, remains open. Whether we agree on how recent governmental policies’ “rhetoric of togetherness may be more seductive than ameliorative” (Sagan and Miller 2018: ii), it seems clear that we need to further enhance and complicate our understanding of loneliness in order to account for its multidimensional nature.

Working at the intersection between psychology and philosophy, Joanna E. McHugh Power, Luna Dolezal, Frank Lee, and Brian A. Lawlor draw attention to the complexity of loneliness, and point out how the focus on social relationships not only “may fail to account for the complex existential, personality, affective, and cognitive aspects of the phenomenon,” but can also overshadow the fact that loneliness as a social construct stems from “largely dysfunctional beliefs about the world, about others, and about the self” (McHugh Power et al. 2018: 223). Using theoretical synthesis methods, their study proposes a framework that integrates research across various disciplines, including psychology, sociology, medicine, and social policy, and argues for the need to further transdisciplinary research in order to offer a more comprehensive definition of loneliness. This opening of the field to other disciplinary perspectives, such as philosophical phenomenology in the case of their study, stems from the conviction that enhancing conceptual elaboration through transdisciplinary collaboration is the first step to improving our understanding of loneliness and, more importantly, bridging the gap between lived experiences of loneliness and current health research definitions of it.

Loneliness scholars insist on how difficult and critical it is to define loneliness and find a suitable language and satisfactory conceptual tools to better describe and address it in all its complexity (Morrison and Smith 2018; Sagan and Miller 2018; Buetow 2023). Yet one of the main challenges is precisely the fact that loneliness is a very private and often stigmatized emotion that is difficult to recognize and to articulate a coherent discourse around. Reflecting on loneliness from the perspective of palliative care, the oncologist Simon Wein acknowledges that “the problem may be in finding the precise words to translate the feeling of loneliness” (2012: 71), and that this can become a real hurdle when trying to establish reliable tools and methods to identify loneliness and distinguish it clinically from other overlapping or competing emotions. Interestingly for our purposes, Wein turns to literary reflections on loneliness by Joseph Conrad, Stephan Zweig, and Aldous Huxley to describe the social, existential, intellectual, political, and moral dimensions of loneliness. These literary insights into what loneliness might be offer eloquent wording of an ambivalent and often conflicting emotional experience and help us overcome the difficulty—some would say the impossibility—of putting embodied experience into words.^3^ In the next section, I will discuss the attempt to do exactly that.

## Narrative approaches

Widely accounted for in the literature of all times, loneliness has been a recurring theme—if not an obsession—in a major strand of contemporary fiction.^4^ Yet only rarely is the literary perspective considered in research on loneliness. There are of course some salient exceptions, like the philosopher and clinical therapist Ben Lazare Mijuskovic’s *Loneliness in Philosophy, Psychology, and Literature* (1979), which makes a case about the innate and pervasive nature of loneliness through the works of classic novelists like Defoe, Conrad, Joyce, Faulkner, and Thomas Wolfe (Mijuskovic 2012). More recently, in her *Biography of Loneliness* (2019), the British cultural historian of gender, emotions, and medicine Fay Bound Alberti uses plays, novels, letters, diaries, and medical case notes from the sixteenth century to the present to historicize loneliness and advance a definition of it as a mutable “emotion cluster.” By putting forward a *longue durée* approach, Bound Alberti disputes the widespread idea of loneliness as something universal and demonstrates not only that loneliness has a history, but that this history changes depending on the meanings attributed to it in different languages and cultures. Although Bound Alberti’s work, like Mijuskovic’s, deals primarily with English texts, she recognizes the need for more investigation in languages other than English (Bound Alberti 2019: 15–16). Also, the fact that the bulk of existing research is based in and focused on English-speaking countries^5^ makes it even harder to develop a more plural, culturally situated framework of analysis, especially if we intend to address loneliness not as a unified phenomenon but as a complex set of emotions with its own and variable set of narrative and linguistic articulations.

If we see loneliness as a multidimensional, historically and culturally situated emotion, then, the stories that we tell around being and feeling lonely constitute an insightful means to better understand a phenomenon that seems to resist any sort of unified approach. My contention is that literature and literary studies can provide a valuable perspective for examining the complexity of loneliness, the meaning and experience of which is variably shaped by the narratives we use to describe what is happening to us. Comparative literature and narrative scholarship in particular can illuminate the ways in which fictional representations solve the challenge of creating a coherent language for the inner experience of private pain related to loneliness and analyze the underlying beliefs, expectations, and preconceived notions that make loneliness so poignant. The contributions of narrative theory to the study of health and care have gained traction over the past years (e.g., Charon 2008; Charon and DasGupta 2011; Charon et al. 2016), and although this research has not yet been directed toward the study of loneliness, its potential is enormous. Introducing perspectives from narrative medicine, comparative literature, and global literary studies into loneliness research would help inform current definitions of loneliness in health scholarship, adding a new layer of analysis, but it would also allow us to perceive loneliness as a constellation of interwoven narratives (fictional and non-fictional, personal, and institutional) that move and circulate at different scales and, of course, beyond the English language.^6^

Broadly understood as meaning-making practices, narratives are powerful forms of expression with well-established effects on healing and well-being (Pennebaker 2000; Pihl et al. 2023; Thornber 2020; Xiang and Yi 2020). The stories that we tell ourselves and others as a form of experiencing and expressing pain or loss, however, are intertwined with myriad other discourses—familial, social, cultural, institutional—that affect one another and ultimately shape our understanding of specific health concerns. As Barbara Sharf, Lynn Harter, Jill Yamasaki, and Paul Haidet point out, “narratives rarely, if ever, have a solitary existence,” and it is the precise purpose of health narrative scholarship to make sense of the contradictions, complications, and uncertainties we find among them (Sharf et al. 2011: 40). This critical function is key because it reveals the diversity of voices and instances at play in the social construction of health and helps deepen our understanding of individual experiences of suffering. Moreover, for scholars like Karen Laura Thornber (2020), narrative theory and literary criticism’s ability to point out and challenge underlying structures of violence and social injustice underscores the importance—and to some extent the obligation—of literary studies to engage with health advocacy and social activism.

To be sure, the use of narrative to illuminate, discuss, and ultimately challenge the underlying dynamics of illness and care can serve to increase social awareness, break down stigmas, and affect health policy. Yet it also raises larger, more difficult questions about how we read and understand these narratives and the type of agency we attribute to them. Writing about the black feminist poet and cancer sufferer Audre Lorde and the revolutionary impact of her *Cancer Journals* (1980), which managed to “translate the silence surrounding breast cancer into language and action” (Lorde 2020: 54), Barbara Sharf reminds us of the power of narratives to influence policy and humanize health care, while also alerting us to the need to “develop more sophisticated criteria for evaluating illness narratives” (Sharf 2001: 218). Sharf is imagining a way of reading that is careful not to reduce illness narratives and personal stories of suffering to their emotional *pathos*. In my view, it is also equally important that, without exaggerating the agency literary texts may have to affect change, our reading is careful not to flatten or dismiss any ambiguities or contradictions some of these texts entail. If literature can be valuable in medical humanities and in health research more broadly, it is precisely as a means of unveiling such ambivalences, rather than as a mere instrument or illustration of a particular argument or cause related to health care, however well-intentioned these may be.

As I discussed at the beginning of this article, the importance of language and narrative as carriers of complex values and meanings proved to be especially relevant during the pandemic, when concerns about “an epidemic of loneliness” became ever more pressing. Soon after the coronavirus forced many of us to shelter in place, rapid research response funds opened specific calls not only for research on controlling the COVID-19 disease, but also for research in the social sciences, the humanities, and the arts addressing the socioeconomic and cultural effects of the pandemic. In the earliest of these initiatives within literary studies, efforts were directed at studying the evolving language of the disease, with the goal of comparing and analyzing the ways in which the pandemic was being narrated in different languages and media across the world.^7^ The archiving of these pandemic narratives and the use of digital mining tools to scrutinize a large corpora of texts allowed researchers to analyze how rhetorical and narrative forms were being deployed to respond to a moment of crisis almost as the pandemic unfolded. But it also spurred interest in collecting and revisiting the large archive of literary works that had tried to make sense of similarly difficult times in the past, leading to timely meditations on the ways these works can help us think about our own pandemic experience, including that of loneliness.^8^

From the very start of the lockdowns across Europe, many readers (and publishers) were quick to turn to novels about plague and confinement—be it on an island or in a hospital, a mental institution, or one’s own body or home. In the UK, the first reports pointed to an unusual burst in sales of classics like Boccaccio’s *Decameron*, Defoe’s *Journal of the Plague Year* and *Robinson Crusoe*, Albert Camus’s *The Plague*, Anne Frank’s *Diary*, Sylvia Plath’s *The Bell Jar*, and Gabriel García Márquez’s *One Hundred Years of Solitude*. The same happened with novels that were written shortly before the pandemic but appeared in a context that made them unexpectedly prophetic, like Ling Ma’s *Severance*, Maggie O’Farrell’s *Hamnet*, Lawrence Wright’s *The End of October*, Fernanda Trías’s *Mugre rosa* (*Pink Slime*), or Emma Donoghue’s *The Pull of the Stars*, among many others.^9^ Despite being a solitary experience, reading novels during the pandemic provided a mix of solace, security, and evasion, while offering a way of dealing with uncertainty and distress and building a sense of belonging and community that helped many readers to face their feelings of loneliness.^10^

In Spain, together with the titles above mentioned, Santiago Lorenzo’s *Los asquerosos* (The Loathsome) became a must-read for many during lockdown. After ten editions and more than 180,000 copies sold, this 2018 novel had a massive and unexpected success, spurred by the pandemic, with recognition from readers and critics, several national awards, and both stage and movie adaptations.^11^ Written in a very peculiar style, with features of orality and a baroque, convoluted voice curdled with wordplay and neologisms, the novel has nevertheless been translated into several languages, including French, Italian, German, and Chinese.^12^ However, the fact that the novel is not available in English—and that it has had limited circulation outside Spain overall—places it in a context that the story seems to reclaim. Set in one of the many fading towns in Spain’s rural, largely unpopulated interior provinces, Lorenzo’s novel reads as a political and lyrical meditation on life in isolation that contains some striking hints of the pandemic that was about to arrive. Diverging from the apocalyptic and dystopian undertones of most pandemic literature, however, the novel thrives by offering a critique of the present that is humorous yet equally pointed.

## Reflecting on loneliness from “Empty Spain”

Advertised as “a static thriller, a version of *Robinson Crusoe* set in empty Spain,” *Los asquerosos* tells the story of Manuel, a rural castaway in one of the thousands of crumbling, depopulated villages in mainland Spain. During the pandemic in particular—but also before it—the reference to Crusoe helped draw attention to the novel, assimilating it to Daniel Defoe’s story of isolation and survival on a remote, deserted island. In a line of continuity that can indeed be traced throughout the novel, *Los asquerosos* came to be praised in a similar fashion to *Robinson Crusoe*, which was commended as “one of the best books to read as we endure the uncertainty and isolation due to COVID-19, because it invites us to reflect on existential issues at the core of a pandemic” (Gunderman 2020). The reasoning behind this and other timely praises of Defoe’s oeuvre during lockdown is substantiated by well-established readings of Crusoe as someone who “more obviously, of course, suffers from loneliness” (Mijuskovic 2012: 26). As we will see, though, loneliness does not in fact feature among Crusoe’s existential worries, as Bound Alberti argues in her *Biography of Loneliness*, and the reflection on isolation and aloneness that we do find in Defoe’s novel adheres to an ideology and set of values that Lorenzo sets out to critique while pondering the ambivalences of solitary life in contemporary rural Spain.

There is a certain tension, then, between the reference to *Robinson Crusoe* displayed in paratexts and interviews with the author and what we find in the pages of the novel, where the only explicit mention of Defoe serves to distance Manuel’s story from “the Crusoes, the Thoreaus, the stylites, the classic survivors, always in need” (Lorenzo 2018: 90).^13^ And yet the correspondence with Crusoe’s story runs unmistakably from the first paragraphs, with references to the origins of the protagonist, his family, and his name: “I was born in the year 1632, in the city of York”/“He was born in Madrid in 1991”; “of a good family, though not of that country, my father being a foreigner of Bremen, who settled first at Hull”/“His father was one who nobody cared about. His mother, who was the same, was my ex-wife’s sister, whom I haven’t seen for I don’t even know how long. He had no other uncles than me”; “I was called Robinson Kreutznaer; but, by the usual corruption of words in England, we are now called, nay we call ourselves and write our name, Crusoe; and so my companions always called me.”/“Manuel is a false name. But I should not give the real one” (Defoe 2003: 3)/(Lorenzo 2018: 2).^14^

Unlike the supposedly autobiographical protagonist of *Robinson Crusoe*, in Lorenzo’s novel we know of Manuel only through his uncle, the sole narrator of the story, who meticulously recounts the phone conversations he manages to periodically keep up with his stranded nephew. The mediation of the uncle’s perspective, limited as it is, adds distance and fallibility to the story, which nevertheless maintains the breadth and love for detail that we find in the minute entries that Defoe’s Crusoe writes in his personal journal. Nevertheless, *Los asquerosos* does have an autobiographical undertone, since Lorenzo’s own life resembles that of Manuel in various ways. A film director, producer, screenwriter, and author of various novels, Lorenzo (Portugalete, Basque Country, 1964) moved in 2012 to a town similar to Manuel’s on the plains of Segovia, in Old Castile. The town has no more than 20 inhabitants, and he lives outside of all public exposure—a position that he makes clear in the novel, which includes a physical, handwritten note from the author in which he clarifies his aversion to social media and any technological form of communication.^15^

The solitary existence that the protagonist leads during most of the 27 brief chapters that organize the novel contrasts with Manuel’s life at the beginning of the story, in Madrid, where he nevertheless feels more alone than ever. Subsisting on precarious jobs with neither friends nor family support apart from his uncle’s, Manuel lives alone in a small, noisy apartment on the bustling Calle Montera, in the city center, longing for some kind of social contact that never comes: “He lived eager to be with people... He really wanted to go out there, be in the company of others and walk around Madrid fooling around a bit, together in a group of nice friends, with mornings of conversation, afternoons of wandering and nights of drinking. But he couldn’t achieve it, to his torture” (Lorenzo 2018: 12).^16^ Manuel’s loneliness—which is rooted in a radical discrepancy between his desired and achieved levels of social relations, to put it in the terms discussed above—fulfills various functions in the story. It serves to portray the character’s behavior and thoughts (Manuel’s endearing worries about making friends and his comically pitiable, almost pathological inability to do so); points to the structural nature of loneliness in a city and a time of crisis (the novel takes place after the Spanish 15-M movement, which catalyzed much of the discontent caused by the 2008 global financial crisis); and also serves as a counterpoint to the loneliness that Manuel is about to experience in the emptied countryside—that sea-less country within the country that journalist and writer Sergio del Molino (2016) has called “Empty Spain.”^17^

Enticed by “what he had heard about large pockets of depopulation and abandoned villages in the northern sub-plateau, the headwaters of the Duero and the Celtiberian Serranía” (Lorenzo 2018: 35),^18^ Manuel flees the city after a rather grotesque fight with a policeman during a protest near his apartment. Uncertain about the state of the officer, Manuel decides to disappear with the help of his uncle in order to avoid prison, in a sequence that clearly references Spain’s 2015 public safety “Gag Law” and the extensive powers it gave to police. He travels north and ends up in one of the thousands of deserted villages in the rural interior provinces of Spain. Alone in an abandoned house in the fictional town of Zarzahuriel, Manuel survives on what little he has brought with him in the car (a phone, some clothes, a razor, some matches); what he finds in the house (an old mattress, a Formica table, an old collection of Austral books); and some basic online shopping that his uncle sends him every week. Like Robinson’s deserted island, Manuel’s town is “an unassisted vestige without a soul, one more of the hundreds and hundreds of them that today remain abandoned in Spain” (36).^19^ And like the resourceful Crusoe, Manuel survives within “a wasteland enclosure” (“un recinto de tierra baldía” 40) cultivating, collecting, sowing, building, warming up, and refreshing himself with what he has on hand—his old, dismantled car being, like Crusoe’s sunken ship, an invaluable source of tools. On the diegetic level, then, Lorenzo elaborates on Manuel’s continual acts of self-making, mimicking that “minute, ordered description of how things are done” that J.M. Coetzee highlights as the best of Defoe’s writing (Coetzee 2001: 20).

Unlike Crusoe, however, and his desire to maximize utility for his own well-being (that precarious island that he manages to turn into profitable land), Manuel thrives in scarcity and a radical austerity, thus inverting—and mocking—the supposed virtues of the *economic man*. In line with the new economic critics and their suspicion of Crusoe as a salutary expression of economic agency and growth (Grapard 1995; Grapard and Hewitson 2011), Lorenzo’s novel rewrites the story of the castaway to imagine an alternative existence, not as an idealized return to rurality, but as an isolated, self-sufficient life based on spareness and a different engagement with place: “Rural lyricism didn’t interest him at all, just as the kid who draws an airplane isn’t interested in aeronautics, or the chemistry of paper, or the physics of the pen, or the philosophy of aesthetics. He never spoke of the ecosophical, georgic, or telluric dimensions of his stay. He just *stayed*” (Lorenzo 2018: 58; italics in the original).^20^ In the emptied town of Zarzahuriel, Manuel discovers that the loneliness that made him miserable in the city has now turned into blissful solitude. After almost a year of confinement, having noticed that his nephew gradually asked for fewer and fewer things, Manuel’s uncle tells us how Manuel “was doing ‘loneliness exams’: scrutinizing and verifying the undulations of his mood once he had been in solitary confinement, to see how he was responding and to see how it was harming him” (102).^21^ Ironically enough, through these loneliness tests, Manuel comes to understand that his clumsiness in establishing affects and relating to others was in fact pointing the way to this new secluded life, which he finds unexpectedly rewarding:

It happened that none of his complete tranquility had to do with people, but rather with their absence. The emancipation of resources he was immersed in paled before the truly powerful independence he had gained, which was affective. He explained to me clearly that his greatest capital was the fact that his need to talk to others had fallen to a minimum. That emancipation was decisive, for it covered everything with freedom and exemption. (101)^22^

The emancipatory feeling that Manuel has in confinement, which he experiences as a freeing exemption from all economic and social duties, leads him to an almost absolute disaffection. In a gradual but extreme turn, Manuel’s detachment also affects his relationship with his uncle, with whom conversations grow increasingly shorter and more and more infrequent. Manuel’s disaffection reaches its highest and most hilarious peak with the arrival of some weekend visitors, an urban family that decides to enjoy rural life by acquiring and taking up residence next to his refuge, putting him in danger of being discovered. On a Friday, the same day that Crusoe finds his first human companion on the island, and every Friday after that, these annoying weekend visitors, whom Manuel starts calling the “mochufas,” ruin his tranquility with their noise, their addiction to screens, their standardized taste, and their medicines and cosmetics for everything. They are “the loathsome” of the novel’s title: a “global and uniform human conglomerate” (127) representative of the worst of our consumerist and digital society.^23^ Manuel (and Lorenzo) predict that a pandemic will come to catch these people unawares, as would indeed happen less than two years after the publication of the novel. “Manuel told me that the unscaffolded ones like the *mochufas*—so polished, with sleepy defenses—would not last long,” the uncle reports. “When the germs came, these weaklings would be the first to be struck down. The more they perfumed their skin, the more they wore death on the surface” (147).^24^

Manuel’s open fight with his loathsome visitors, which injects moments of true biting comedy throughout the second part of the novel, carries the story to its climax, pushing it into the terrain of parable. Grounded in the eccentric and dubious example of a young man whose indigence and seclusion lead him to almost disappear altogether, Lorenzo’s novel is a celebration of solitude and closeness to nature—in this case, the desolate Castilian countryside. The book’s response to the aberrations of our global society, represented by the titular “loathsome,” is that in solitude, personal and moral degradation does not matter, because they cannot harm anyone. This is an ironic answer, of course, and not exempt from ambiguity. To a certain extent, it is also an easy answer, since it locks itself into a radical individualism that says little, if anything, about how to live in community. More than a collective response, indeed, what Manuel offers is an individual way out of a problem that remains unresolved. And yet his story invites us, rather unpretentiously, to think about the political potential of feelings such as loneliness or disaffection. It does so by reminding us, without any sort of embellishment or idealization, of the positive and salutary dimension of loneliness—the freedom and exemption that Manuel finds in solitude—and also by presenting loneliness not as an individual failure, as we saw most governmental rhetoric implies, but as a bigger, structural problem affecting the city and the country as spaces that are differently subjected to global pressures and the abandonment of the State.

Representing loneliness as an ambivalent experience of blissful tranquility and radical disaffection, Lorenzo’s novel questions any direct causation between loneliness and aloneness or social isolation. And, by redirecting our attention toward the many ills, local and global, that lead the protagonist to almost dissolve into nature, it shifts the focus away from any individual guilt or responsibility of care and onto a more political reading of loneliness. A reading like this is different from the definitions of loneliness as a subjective and relational feeling, and from the language of crisis related to loneliness that is prevalent, as we have seen, in public health literature, government policy, and the media. In contrast, as I have argued, a narrative approach to loneliness that benefits from literary insights like the one analyzed here can complicate our understanding of loneliness and help us reclaim its political potential. While novel reading can indeed offer comfort and entertainment, as Lorenzo’s novel certainly did for many readers before, during, and after lockdown, it can also put our assumptions into question and make us reconsider whether loneliness is a social illness or the symptom of an ill society.

## Data Availability

Not Applicable.

## End notes

^1^ In *The New York Times*, *Harvard Magazine*, and *The Irish Times*, respectively.

^2^ After the UK, in 2021, Japan followed the lead with the creation of a Loneliness Ministry amid the pandemic, with increasing suicide rates, especially among women, as well as solitary deaths (*kodokushi* in Japanese).

^3^ See Andrews (2018) for an excellent discussion of art’s potential to help us reflect on how loneliness is performed in relation to place in the context of climate change.

^4^ See Ferguson (2013) and Majorano (2012) for extensive analyses of loneliness in contemporary North American and French narrative fiction, respectively.

^5^ The lack of data collection in many regions in the Global South is an acknowledged limitation of current empirical research on loneliness. There are no global rates of loneliness and very few studies have been conducted in low- and middle-income countries, where loneliness remains understudied, or not addressed at all. For a summary of available data, see the World Health Organization’s last advocacy brief (WHO 2021).

^6^ For a decentering of the Anglocentric focus in the relatively new field of medical humanities, see Karen Laura Thornber’s *Global Healing: Literature, Advocacy, Care* (2020), which offers critical readings of an impressive array of literary texts from around the world that deal with diverse experiences of illness and adverse health conditions including leprosy, AIDS, Alzheimer, and death.

^7^ Some of these projects include the “Worldmaking in the Time of COVID-19” project, funded by the King’s College London Coronavirus rapid response fund; the Stanford Literary Lab’s “Writing about Epidemic” projects (“The Evolving Language of Disease,” “Literature of Confinement,” and “Personifying Illness”); the “Culture and Quarantine” hub, hosted by the British Comparative Literature Association; the “Narrative responses to the pandemic” project of the Dulwich Centre in Adelaide, Australia; and the Iber-Lab’s COVIDTECA of the University of Granada in Spain (Gallego Cuiñas and Pérez Tapias 2022). For a translational and multilingual perspective on the languages of COVID-19, see Blumczynski and Wilson (2022).

^8^ See the 2022 double special issue on “World Literature In and For Pandemic Times,” edited by David Damrosch, in the *Journal of World Literature* (vol. 7, issues 1 and 2), especially Delia Ungureanu’s article “The Value of Solitude,” which reflects on the revolutionary potential of solitude from antiquity through the works of Montaigne, Shakespeare, and Virginia Woolf.

^9^ For some figures, see Flood (2020).

^10^ For an ethnographic study about the ways novel reading changed during the COVID-19 pandemic in Denmark and the UK and the ways it emotionally impacted readers, see Davies, Lupton, and Gormsen Schmidt (2022). On reading as a social and relational activity, see Fuller and Rehberg Sedo (2013) and Birke (2021).

^11^ When it first came out in 2018, *Los asquerosos* won the Spanish Cálamo Award for Book of the Year and the Booksellers Recommend Award, granted by the Spanish Confederation of Booksellers, which brings together more than 1500 bookstores throughout Spain. The theater piece, directed by David Serrano, premiered in December 2020 in Madrid, and two years later it was the subject of an audio movie by the same director (Sonora, 2022).

^12^ I thank Jan Martí, founder and editor of Blackie Books, for providing details about sales figures and translation rights, which have been sold to Italy (*Gli schifosi*, Blackie Edizioni, 2020), Taiwan (ACME Publishing, 2020), France (*Les dégueulasses*, Editions du Seuil, 2021), Germany (*Wir alle sind Widerlinge*, Heyne Hardcore, 2022), and Portugal (Gradiva Publications).

^13^ All translations of Lorenzo’s novel are my own, with the invaluable help of John Shakespear: “No se le veía muy asimilado a los Crusoe, a los Thoreau, a los estilitas, a los supervivientes clásicos, siempre menesterosos.”

^14^ “Nació en Madrid en 1991. Su padre era uno que le daba igual a todo el mundo. Su madre, que lo mismo, era la hermana de mi exmujer, a la que no veo desde hace ya ni sé. No tenía más tíos que yo. Impresionaba verle, con once años, buscando trabajo en Internet. No se lo iban a dar ni él lo iba a pedir, por su edad. Pero desde crío, Manuel ya estaba indagando sobre cómo sería verse a sí mismo metido en el mundo. Manuel es un nombre falso. Pero es que no debo dar el verdadero.”

^15^ To learn more about the author, see the interviews by Elorduy (2018), Ligero (2019), Gosálvez (2019), and Hernando (2022).

^16^ “Vivía ávido de tratar con gente… Tenía muchas ganas de ir por ahí, de salir en compañía y de andar por Madrid haciendo un poco el gamba, engarzadito en un grupo de amigachos majos, con mañanas de conversación, tardes de callejeo y noches de vasos. Pero no se le lograba, para tortura suya.”

^17^ On the concepts of empty/emptied Spain, and from a gender perspective, see also Virginia Mendoza’s *Quién te cerrará los ojos. Historias de arraigo y soledad en la España rural* (2017) and bestselling María Sánchez’s memoir *Tierra de mujeres* (2019), translated as *Land of Women* in 2022.

^18^ “Tiró hacia el norte, inducido por lo que tenía oído sobre grandes bolsas de despoblación y aldeas abandonadas en la submeseta septentrional, la cabecera del Duero y la Serranía Celtibérica.”

^19^ “un vestigio desasistido y sin un alma, uno más de los cientos y cientos de ellos que hoy permanecen abandonados en España.”

^20^ “La lírica agreste no le interesaba nada, como al crío que dibuja un avión no le interesa ni la aeronáutica, ni la química del papel, ni la física del bolígrafo ni la filosofía de la estética. Nunca hablaba de la dimensión ecosófica, ni geórgica, ni telúrica de su estancia. Se limitaba a *estanciar*.”

^21^ “Me contó que hacía ‘exámenes de soledad’: escrutar y verificar las ondulaciones de su ánimo una vez sometido a la incomunicación, a ver cómo estaba respondiendo y a ver cómo le estaba perjudicando.”

^22^ “Le estaba pasando que nada de su plena tranquilidad tenía que ver con las personas, sino con la ausencia de ellas. La emancipación de recursos en la que vivía inmerso palidecía ante la independencia realmente poderosa con la que se había hecho: la afectiva. Me explicó a las claras que su gran capitalazo radicaba en que tenía bajo mínimos la necesidad de pegar la hebra a nadie. Esa manumisión sí que era decisiva, esa sí que lo pringaba todo de libertad y de exención.”

^23^ On the use of new technology and AI to reduce loneliness and social isolation, see Tkatch et al. (2021), Grey et al. (2024), and Zheng et al. (2023). For a meditation about the effects of technology in our social lives, see Turkle (2012).

^24^ “Apuntaba Manuel que los desandamiados como los mochufas, tan pulimentaditos, con defensas adormiladas, durarían poco. Cuando viniera el germen, estos enclenques serían los primeros derribados. Llevaban la muerte más a flor de piel según más se perfumaban el cutis.”
